# A Novel Fabric Strain Sensor Array with Hybrid Deep Learning for Accurate Knee Movement Recognition

**DOI:** 10.3390/mi17010056

**Published:** 2025-12-30

**Authors:** Tao Chen, Xiaobin Chen, Fei Wang

**Affiliations:** 1 School of Future Technology, South China University of Technology, Guangzhou 511422, China; 202310193058@mail.scut.edu.cn; 2 Research Institute for Intelligent Wearable Systems, The Hong Kong Polytechnic University, Hong Kong 999077, China; xiaobin23.chen@connect.polyu.hk; 3 School of Fashion and Textiles, The Hong Kong Polytechnic University, Hong Kong 999077, China; 4 School of Textile Science and Engineering, Wuyi University, Jiangmen 529020, China

**Keywords:** fabric strain sensor, knee joint monitoring, sensor array, wearable electronics, deep learning, CNN + BiLSTM, attention mechanism, posture classification

## Abstract

This paper presents a novel lightweight fabric strain sensor array specifically designed for comprehensive knee joint monitoring. The sensor system features a unique two-layer design incorporating eight strategically positioned sensing elements, enabling effective spatial mapping of strain distribution across the knee during movement. This configuration offers advantages in capturing complex multi-axis kinematics (flexion/extension, rotation) and localized tissue deformation when compared to simpler sensor layouts. To evaluate the system, ten subjects performed three distinct activities (seated leg raise, standing, walking), generating resistance data from the sensors. A hybrid deep learning model (CNN + BiLSTM + Attention) processed the data and significantly improved performance to 95%. This enhanced accuracy is attributed to the model’s ability to extract spatial-temporal features and leverage long-term dependencies within the time-series sensor data. Furthermore, channel attention analysis within the deep learning model identified sensors 2, 4, and 6 as major contributors to classification performance. The results demonstrate the feasibility of the proposed fabric sensor array for accurately recognizing fundamental knee movements. Despite limitations in the diversity of postures, this system holds significant promise for future applications in rehabilitation monitoring, sports science analytics, and personalized healthcare within the medical and athletic domains.

## 1. Introduction

Wearable electronic sensors have revolutionized the field of healthcare [1], sports science [2], and personalized medicine [3] by enabling continuous monitoring of physiological and biomechanical parameters. Among these, knee joint monitoring is crucial for rehabilitation support, injury prevention, and chronic disease management, as it offers precise measurement of movement angles, forces, and load distribution [4]. Knee joint monitoring systems use wearable sensors to capture detailed kinematic and kinetic information during daily activities, enabling accurate assessment of gait patterns and joint stability. When combined with modern soft materials, these devices offer comfortable long-term use, real-time feedback, and personalized rehabilitation or injury-risk predictions.

Early studies in knee joint monitoring deployed traditional rigid sensors, such as Inertial Measurement Units (IMUs) [5] and optical systems [6]. However, they face limitations in terms of comfort, seamless integration, and long-term durability, often leading to motion artifacts and environmental interference that compromise measurement accuracy [7]. IMU sensors are rigid and offer little comfort. The camera-based systems commonly used for human motion capture combine optical imaging data and computer modeling analysis. They can monitor movement with high precision but are confined to laboratory settings, as they cannot be worn and the systems are cumbersome [8]. Other non-textile flexible sensors face challenges in knee joint monitoring: hydrogel strain sensors [9,10] struggle with garment integration and are non-washable; skin-adhesive patches and epidermal electronics [11,12] may cause skin irritation with prolonged wear; thin-film strain gauges [13] have limited strain measurement ranges. Moreover, flexible sensors often require frequent recalibration, and their performance can be influenced by factors like temperature, humidity, sweat, and mechanical wear, limiting their consistency over time [14]. For instance, a review on fabric-based sensors highlights that “accuracy, reliability, calibration, durability, interference, cost, and compatibility” are the key challenges that need to be overcome [15].

Recently, fabric-based sensors have emerged as a promising alternative due to their flexibility, comfort, and adaptable ergonomic integration into clothing [5], making them ideal for long-term knee joint monitoring. For example, Shyr et al. developed a textile-based wearable sensing device for monitoring the flexion angles of elbow and knee movements via a strain-sensitive textile placed over the joint region [6]. More recently, Galli et al. presented a fully textile capacitive sensing system for knee angle monitoring, achieving strong correlation (R^2^ up to ~0.99) with optical motion capture in preliminary tests [8]. These works collectively show that fabric-based sensors conform to the previously stated wearability advantages (flexibility, garment integration, comfort) and that they have been preliminarily evaluated in knee joint or general joint motion monitoring.

Despite the above advances, fabric-based sensors for knee monitoring still face significant limitations: calibration drift and hysteresis remain concerns, particularly under repeated stretching and washing cycles [9]; sensor placement and movement relative to the skin/garment (slippage) can degrade accuracy; the strain or angle range may still be limited for high-intensity or complex movements (e.g., pivoting, lateral cut) and integration into truly everyday wearable garments (with laundering, sweat, multi-user variability) is still nascent [8].

Therefore, it can be seen that fabric-based strain sensors have emerged as a promising solution for knee joint motion monitoring, but they remain limited in their current form. One typical limitation is that these sensors provide valuable data on strain and angle changes at specific points of the knee, but they are often constrained by their lack of spatial coverage [8]. Most studies [16] on fabric strain sensors place a single sensor over the knee, which fails to provide a comprehensive view of the strain distribution across the entire joint surface. While using multiple sensors arranged in an array could offer a more complete picture, this approach introduces complexities, such as managing the intricate wiring and signal processing of multiple sensors [8,16,17].

The dual-layer FSS (Flexible Strain Sensor) array [18] developed previously in our research group, however, represents a breakthrough, incorporating built-in wiring within the array itself, which simplifies the setup and offers an advanced solution for continuous, multi-point monitoring of knee movement. The flexible printed fabric strain sensors offer distinct advantages, rendering them suitable for both clinical and sports applications. These sensors can be readily integrated into clothing or wearable devices, offering lightweight properties alongside a substantial strain measurement range. They enable long-term monitoring of joint movements, such as the dynamic changes in the knee during exercise. Their suitability for mass production and cost-effectiveness enhance their potential for everyday applications, presenting an exciting new avenue for future research and practical deployment.

However, despite their great potential, these fabric-based sensors still face challenges regarding their ability to provide precise, real-time feedback and accurate long-term monitoring without the support of advanced data processing techniques. Without algorithms to process sensor data effectively, fabric-based sensors lack the ability to offer real-time analytics, predictive insights, or detailed movement analysis, which limits their applicability in clinical and sports environments. It is worth noting that the integration of artificial intelligence (AI) and machine learning (ML) algorithms showed great potential to overcome these limitations [1]. In various fields, AI has already shown immense potential in enhancing sensor-based technologies [19], such as improving the accuracy of heart rate monitoring [20], gait analysis [21], and injury prediction [22]. For example, deep learning models like Convolutional Neural Networks (CNNs) and Long Short-Term Memory networks (LSTMs) have been successfully applied in gait analysis to detect steps, classify walking patterns, and even predict fall risks [23]. Similarly, edge computing is being leveraged to process sensor data locally on wearable devices, providing real-time feedback without relying on cloud computing [24]. These AI and data processing technologies can enhance the capabilities of fabric-based strain sensors by enabling precise strain distribution mapping across the knee joint, improving the accuracy of motion analysis, and providing immediate feedback during activities. Furthermore, by analyzing long-term sensor data, AI can offer predictive insights into joint health and injury risks, making it a valuable tool for both rehabilitation and athletic performance [25]. Therefore, the combination of FSS arrays with AI-driven algorithms will not only address the hardware limitations of fabric-based sensors [16,26,27,28,29,30,31,32,33,34,35,36,37,38,39] but will also significantly improve their functionality [4,40,41,42,43,44,45,46], providing more accurate, dynamic, and actionable data [47,48,49,50,51,52] for knee joint monitoring [53,54,55,56,57,58,59,60,61] in real-world, long-term applications. Furthermore, the research into the application of wearable sensors and ferroelectric nanogenerators in the fields of sports [62] and medicine [63] is continuously expanding. Such exploration serves as a guarantee for obtaining more accurate and reliable data [64] in practical engineering applications.

In this regard, this paper introduces this novel lightweight fabric strain sensor array with a two-layer design and eight strategically placed sensing elements for comprehensive knee joint monitoring, enabling effective spatial mapping of strain distribution during movement. A hybrid deep learning model (CNN + LSTM + Attention) achieved 95% accuracy by extracting spatial-temporal features and leveraging long-term dependencies in sensor data, with channel attention analysis highlighting sensors 2, 4, and 6 as key contributors. Despite posture diversity limitations, the system demonstrates promise for rehabilitation monitoring, sports analytics, and personalized healthcare applications.

## 2. Materials and Methods

### 2.1. Design and Fabrication of Knee Fabric Sensor

**Figure 1:**
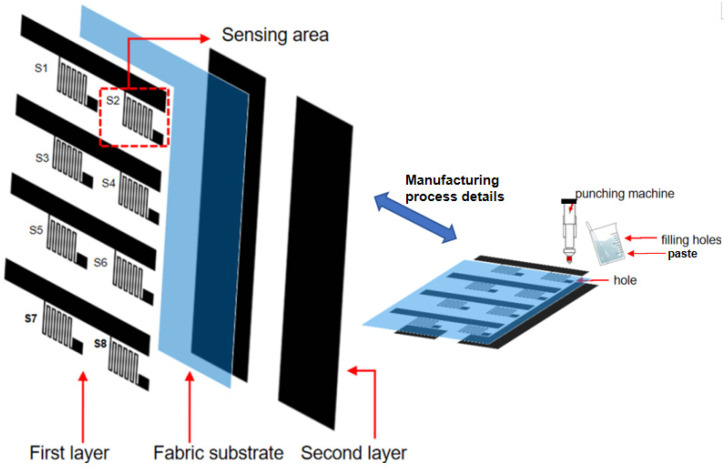
Schematic of the fabric strain sensor (FSS) and its three-layer structure. The interconnect layer on the second layer is electrically connected to the sensors on the first layer via vertical vias. The strain sensing mechanism is mainly the tunneling effect of the CB/SE conductive composite materials. The same material was used to fill the small via holes as a paste.

Traditional knee sensors typically use multiple sensor units from different categories, while this knee sensing system we designed only requires two layers, i.e., upper and lower layer design to make a fabric strain sensor array. For specifics on the sensor preparation process, refer to previous works [18,65]. The design of knee fabric sensor is shown in Figure 1. The sensor array includes 8 strain sensing elements arranged in two columns.

The first layer functions as the strain sensing element, typically composed of a conductive composite coated onto a fabric substrate, which directly transduces mechanical deformation into measurable electrical signals. The substrate layer, constructed from an elastic and breathable knitted fabric, ensures conformal contact with the knee surface, thereby maintaining user comfort during prolonged wear. The second layer is made from the same conductive material, electrically connecting rows of strain sensing elements. This two-layer configuration not only simplifies the fabrication process compared to conventional multi-unit sensor arrays but also enhances mechanical flexibility and facile integration with wearable garments. As a result, the proposed knee fabric sensor achieves a lightweight, low-profile design capable of reliable and repeatable performance. Furthermore, this configuration not only minimizes interfacial mismatches and assembly steps but also facilitates large-area production through scalable textile manufacturing techniques such as screen printing, dip coating, or electrospinning. Furthermore, by eliminating the need for rigid sensor modules or multiple heterogeneous sensing units, the device achieves a thinner profile, lighter weight, and higher integration with wearable garments. Therefore, such a streamlined structure is particularly advantageous for dynamic knee monitoring in sports, rehabilitation, and daily activity tracking, where unobtrusiveness, repeatability, and durability are essential for both user compliance and measurement reliability.

The materials and fabrication process of this fabric strain sensor followed our previous work [18,65], and the details are presented as follows. The conductive composite was prepared by uniformly mixing carbon black (3.0 g), room-temperature vulcanizing silicone rubber (30.3 g), and silicone oil (45.0 g) in a plastic beaker, followed by stirring in a blender at 400 rpm for 30 min. The substrate material is a plain-knit fabric composed of 70% polyester fiber and 30% Lycra fiber. To form the conductive pattern, the composite was screen-printed onto both sides of the substrate, and vertical interconnect access (vias) were punched at designated positions and filled with the composite to establish electrical connections between the two sides. The final fabric sensor array was obtained after curing in an oven at 100 °C for 1 h. The mixer used in sensor manufacturing was purchased from Shanghai Lichen Instrument Technology Co., Ltd. (Shanghai, China). The printing machine used in sensor manufacturing was purchased from Dongguan Deliou Precision Equipment Co., Ltd. (Dongguan, China).

The sensor comprises a single continuous conductive pattern printed in one step, which integrates two functional regions: sensing part and connection part. In the sensing part is a meandering trace with an effective length of 221 mm and a width of 2 mm, designed to achieve a high aspect ratio (>110) and thus a high initial resistance for strain sensing. The connection part is designed as wider rectangular areas (20 mm × 20 mm) with an aspect ratio of 1, providing a low-resistance path for reliable electrical interfacing.

Notably, the sensor array is made from a uniform composite material, in which a high loading of carbon black particles leads to the formation of aggregates that constitute the conductive network within the silicone elastomer matrix. Electrons can tunnel through the junctions between adjacent carbon black aggregates when the inter-aggregate distance is sufficiently small. Under strain deformation, this distance increases, resulting in reduction in electron tunneling probability. When a readout circuit is connected across the electrodes, this phenomenon manifests as a change in electrical resistance.

Considering the size of the knee and the actual wearing experience, the design of 8 sensors can enable more accurate monitoring and recognition of lower-limb movement postures. To address its complex multi-axis movements (primarily flexion/extension, accompanied by internal/external rotation and adduction/abduction), the sensor array is designed uniaxially along the distal direction to be able to precisely capture joint angle changes as well as to monitor patellar trajectory and tibiofemoral joint contact force distribution. Considering the critical role of ligaments (such as the anterior cruciate ligament (ACL), posterior cruciate ligament (PCL), medial collateral ligament (MCL), and lateral collateral ligament (LCL)) in stabilizing the joint and transmitting loads, strain sensors are integrated to non-invasively assess their tension state.

In our design, both the sensing and connecting sections are fabricated using the same carbon black/silicone elastomer (CB/SE) composite material via a single-step screen printing process. The key to ensuring the connecting section does not interfere with the strain response lies in the specifically designed aspect ratio (length/width) which was chosen meticulously.

The initial resistance R_0_ of the conductive tracks follows Ohm’s law, R ∝ L/W, where L and W denote the length and the width of the conductive film. The sensing section employs a serpentine structure (effective length = 221 mm, width = 2 mm, aspect ratio > 110), while the connection section features an extremely low aspect ratio design (length = width = 20 mm, aspect ratio = 1). This results in R_s0_ ≫ R_c0_, meaning the initial resistance of the sensing section dominates the total resistance value. Consequently, when under strain, the relative resistance change ΔR/R_0_ is primarily contributed by the sensing section, while the connection section functions primarily as a low-resistance interconnect structure. Therefore, the resistance change in the connection part can be reasonably neglected in the overall resistance change.

The eight sensors in the first layer are divided into four rows, with two sensors in each row, and they are numbered: Sensor 1, Sensor 2, and so on, as illustrated in Figure 1. The design of the second layer is intended to electrically connect the sensors in the first layer. An array of eight sensors can simultaneously measure the magnitude of strain at each position, enabling more detailed measurements within the covered surface area. In this way, the system can generate a detailed spatial strain map. This mapping capability is crucial for obtaining comprehensive bio-mechanical characteristics of joint movement, tissue deformation, or applied force, revealing subtle differences that single-point sensors may overlook. Additionally, a key aspect of our analysis is to investigate the relative contribution of each sensor to the overall feature set. In subsequent studies, we will present a detailed feature importance analysis. This analysis will quantify and rank the signal importance of sensors 1 to 8 in characterizing the monitored phenomenon (e.g., specific joint angles, gait phases, or pathological states). Mapping this feature importance distribution is of critical significance: it empirically validates the functional advantages of our specific fabric sensor design and layout strategy. By identifying which sensors provide the most discriminative or reliable information, we can demonstrate the effectiveness of the array configuration in capturing relevant bio-mechanical features and may provide a basis for optimizing sensor placement in future knee joint monitoring applications.

### 2.2. Data Collection and Experimentation

Ten male subjects were recruited for the test. Before participating in the test, the subjects provided their informed consent. The test included three parts, in which the subjects sit and lift their legs, stand, and walk. Table 1 presents the height, weight, and age information of the subjects.

In total, this test resulted in 30 experimental trials per movement category, providing a diverse dataset that captures inter-individual variations in knee joint dynamics as well as intra-individual variability across repeated sessions. Additionally, the dataset captures inter-individual variability within repeated testing sessions, which may stem from factors such as fatigue, environmental conditions, or subtle postural differences. This comprehensively illustrates the knee joint’s behavioral characteristics under varying conditions. By integrating both inter- and intra-individual variability, the dataset is suitable for training artificial intelligence models that require both cross-user generalization capabilities and sensitivity to fluctuations within an individual’s repeated performance.

**Figure 2:**
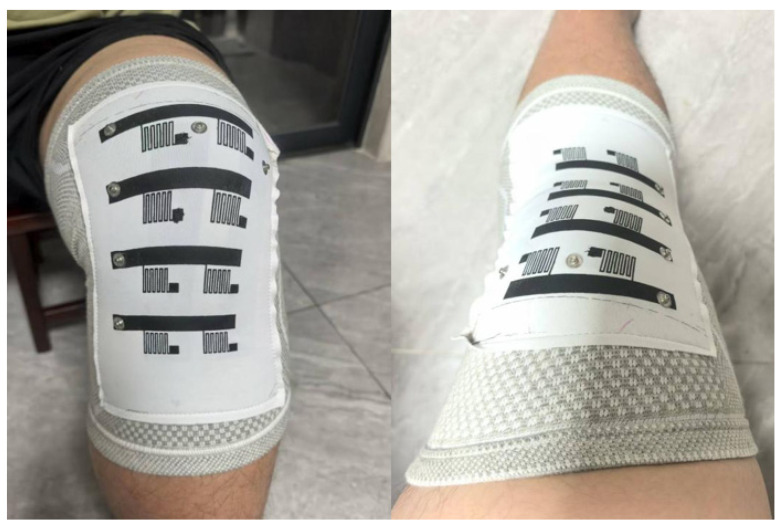
Typical images during the test. Lifting the leg (**right** picture) and bending the knee (**left** picture) while sitting with a fabric sensor knee pad attached.

As given in Figure 2, the experimental process is: when the subject first put on the knee pads, the location of the fabric strain sensor will be adjusted to make sure the knee is fully covered. Also, as peripheral of the sensor array were sewn onto the elastic fabric kneepad before the experiment, the two ends of the sensor array can maintain their relative position to the thigh and the calf, respectively.

Supplementary instructions for each movement are as follows: Kick: In the experiment, the kick movement must be performed within a range starting from a 90° knee bend and extending outward to approximately 180° (i.e., nearly fully straightened with slight hyperextension). Each participant performs 5 repetitions. The rhythm control for each movement is as follows: approximately 2 s to lift the leg, approximately 2 s to return to the starting position (each complete cycle takes about 4 s), with a 15 s rest between sets. If the subject feels discomfort, the angle or number of repetitions may be reduced. Standing Task: Participants maintain a natural standing posture for 1 min (with only minor adjustments to position, avoiding movement as much as possible) to assess sustained weight-bearing and postural control. Walking Task: Continuous for 1 min. For quantification and comparison with subsequent results, the common normal walking speed is estimated at approximately 1.2 m/s (about 72 m/min), corresponding to a step frequency of approximately 100 steps/min.

After each posture test, a CSV (Comma-Separated Values) file of 8 sensor resistance values will be generated, which will be saved in time to do the next action posture, until the data collection of three actions is done. To ensure consistency, each action is performed five times by every tester, and the corresponding sensor outputs are systematically recorded. The collected data are then organized into structured datasets, labeled according to the action category, test, and trial sequence, thereby facilitating subsequent data preprocessing, model training, and performance evaluation.

### 2.3. Posture Examples and Corresponding Resistive Line Diagram Results

**Figure 3:**
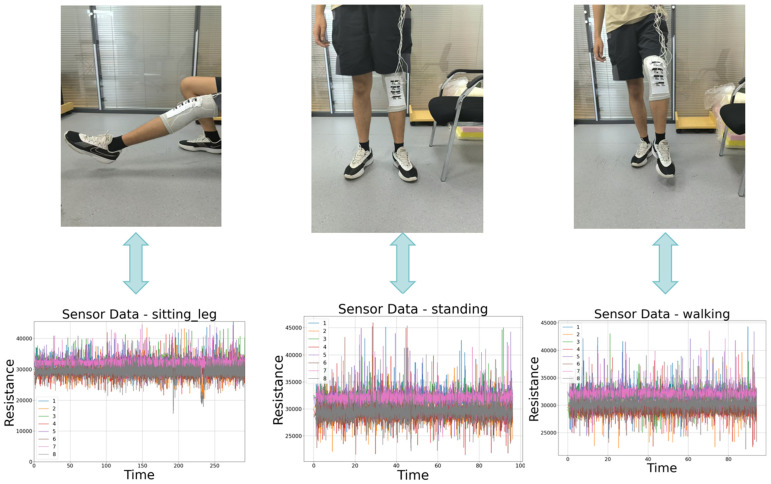
Examples of 3 postures and corresponding resistance line graphs.

During the experiment, we found that the most significant resistance change occurred during the sitting and lifting motion, followed by walking, and then standing. As illustrated in Figure 3 below, the three distinct movements correspond to three separate resistance variation curves. Each action exhibits corresponding differences in the amplitude, rate of change, and waveform characteristics of the resistance response, which align precisely with the actual movement sequences. This provides direct verification of the sensor’s accuracy and reliability in motion recognition. By comparing the morphological characteristics of the curves, flexion-extension, rotation, and lateral sway actions can be clearly distinguished. This demonstrates that the sensor not only captures strain signals generated by joint movements in real time but also maintains highly consistent response characteristics across multiple repeated experiments. This further validates its stability and repeatability in motion monitoring and pattern recognition.

To ensure consistency of sensor attachment during the study, we employed a controlled experimental procedure. The fabric strain sensor array was carefully positioned on each subject’s knee and adjusted to ensure complete coverage of the knee joint. To maintain consistent sensor placement, the array was sewn onto an elastic fabric knee pad, ensuring that the upper and lower boundaries of the array remained fixed relative to the thigh and calf, respectively, during movements. Data from each trial was systematically recorded, with repeated testing ensuring that any discrepancies in sensor data were due to the movement itself, rather than sensor placement issues. Furthermore, we plan to continue evaluating the sensor’s long-term attachment stability in future tests.

Furthermore, we standardized the donning process, conducting visual and tactile checks and initializing standardized experimental postures before each experiment. When each subject wore the knee brace, we ensured that: the center hole of the knee brace was aligned with the center of the patella; the upper edge of the knee brace was aligned with the lower part of the thigh, and the lower edge was aligned with the upper part of the calf; and the longitudinal axis of the sensor array was substantially parallel to the flexion-extension axis of the knee joint.

### 2.4. Posture Triple Classification Model and Its Prediction

We processed the data for 3 individual files and all 30 files in each of the three categories. Random forest algorithm was used to carry out the construction of multiple decision trees to combine the results for categorization, for different categories of CSV files (one CSV file was generated for each detected action to record the resistance value of each sensor), which were categorized as sitting and lifting the leg, standing, and walking. The work of feature extraction, model training, model prediction and model evaluation was carried out.

The CNN (Convolutional neural network) + BiLSTM (Long short-term memory network) + Attention hybrid deep learning model architecture combines the local feature extraction capabilities of CNN, the time series modeling capabilities of BiLSTM, and the feature selection capabilities of the attention mechanism. It is specifically designed for time series sensor data classification tasks.

**Figure 4:**
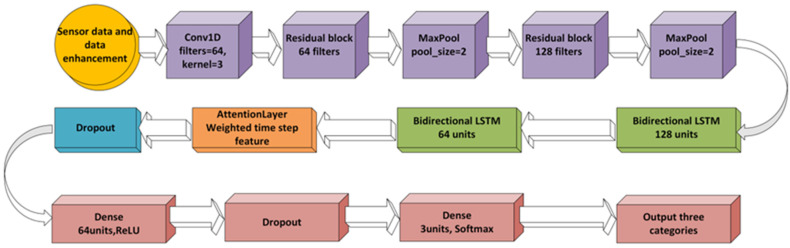
Convolutional neural network (CNN) + Bidirectional long short-term memory network (BiLSTM) + attention mechanism deep learning model, specially designed for three types of posture and movement recognition.

As shown in Figure 4, the CNN part extracts local spatial-temporal features, the BiLSTM part captures long-term temporal dependencies, and the Attention part dynamically focuses on key time steps (such as key signal segments). This architecture balances feature extraction capabilities (CNN), temporal modeling capabilities (BiLSTM), and feature selection capabilities (Attention). By integrating these three components into a unified framework, the model achieves a more comprehensive representation of the sensor data, effectively bridging the gap between low-level signal fluctuations and high-level motion patterns. The CNN ensures that subtle variations in resistance signals caused by localized joint movements are preserved, while the BiLSTM leverages sequential dependencies to model the continuity of motion across time. The Attention mechanism further refines this process by weighting the most informative temporal segments, thereby filtering out redundant or noisy inputs. As a result, the architecture not only improves recognition accuracy under standard testing conditions but also demonstrates enhanced robustness against motion artifacts, signal drift, and inter-subject variability.

This hybrid architecture, combining CNN, BiLSTM, and Attention mechanisms, offers a holistic approach to sensor data analysis by efficiently capturing both the spatial and temporal dynamics of knee joint movement. The CNN component is particularly adept at extracting localized features from the raw sensor signals, enabling it to detect minute but significant variations in sensor resistance that correspond to small joint movements, such as flexion or extension. This step ensures that the fine-grained details of motion are preserved and utilized in the subsequent analysis.

The BiLSTM layer, on the other hand, excels at capturing the temporal dependencies between consecutive sensor readings, allowing the model to model the continuity of movement over time. By maintaining memory of past observations, the BiLSTM can learn long-range dependencies within the data, which is crucial for understanding cyclic or repetitive motion patterns, such as walking or squatting, where each phase of movement influences the next.

The Attention mechanism enhances this process by focusing the model’s computational resources on the most relevant segments of the input data, dynamically assigning higher weights to time steps that contain critical features for classification. The integration of these three components in a unified framework enables the model to handle complex knee motion data with high precision.

## 3. Results

### 3.1. Results After Processing 3 CSV Files with a Random Forest Algorithm

**Figure 5:**
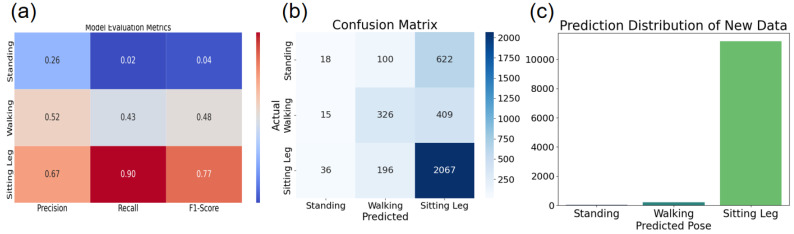
(**a**) Model evaluation metrics: comparison of specific values of precision, recall, and F1 score in three poses; (**b**) Confusion matrix results for three different poses; (**c**) An example of the results predicted by the new data.

The results generated using the random forest algorithm are as follows:

The model evaluation including precision, recall, and F1 score is shown in Figure 5a. The confusion matrix is shown in Figure 5b. Among the results of the visualization, the confusion matrices for the three different postures, the results of which show that the model recognition performs best in the posture of sitting and lifting the leg. For example, if there is new data appearing, the features of the 8 sensors can be predicted more accurately with the Random Forest classifier, as shown in Figure 5c. The prediction of the new data has a high probability of sitting and lifting the leg.

### 3.2. Results for All 30 CSV Files with Deep Learning Model

When using CNN + BiLSTM + Attention for the same prediction, the accuracy rate was 95%. This is partly due to differences in data volume and partly due to differences in model architecture complexity. Therefore, the results visualization under the deep learning model shows an improvement in performance optimization.

**Figure 6:**
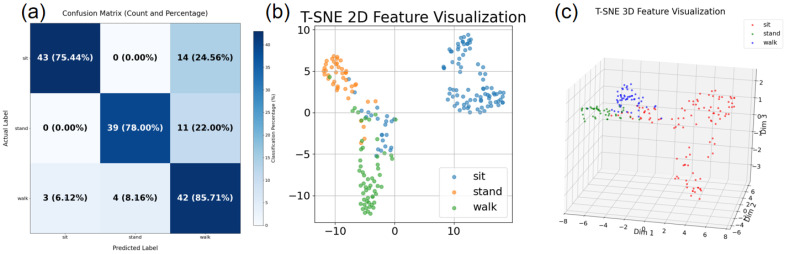
(**a**) Confusion matrix results after training; (**b**) T-SNE 2D feature visualization; (**c**) T-SNE 3D feature visualization.

As shown in Figure 6, the three images reveal the advantages of deep learning models from different perspectives. The observed improvement in accuracy highlights the significant impact of architectural optimization and data scale on model performance. Convolutional neural networks (CNNs) [10] are renowned for their ability to effectively extract spatial features from input data, particularly in image and sequence-related tasks [11]. By integrating bidirectional long short-term memory (BiLSTM) units, the model gains the ability to capture temporal dependencies and sequence patterns that pure CNN models may overlook. This is particularly important in scenarios where data has time series or ordered features. Additionally, the introduction of attention mechanisms enables the model to dynamically focus on the most relevant parts of the input sequence, assigning higher weights to important features during the learning process. This combination enhances the model’s ability to learn complex patterns and relationships in the data.

Differences in the amount of data between models play a key role in driving performance improvements. Larger datasets typically provide models with more diverse and rich learning information, helping to reduce overfitting and improve generalization capabilities on unseen data. When data is limited, even the most complex model architectures may struggle to achieve optimal performance due to insufficient training samples. Conversely, increasing the quantity of high-quality training data enables models to better fit the underlying data distribution, leading to more robust prediction results. Optimization strategies and training protocols also influence final accuracy. Techniques such as learning rate scheduling, batch normalization, dropout, and early stopping significantly impact model convergence and generalization capabilities [12]. Reasonably adjusting these hyperparameters typically enhances model stability and prevents it from getting stuck in local optima or overfitting noise. Additionally, the choice of loss functions and evaluation metrics directly influences training priorities and reported performance outcomes.

In addition to data volume and standard optimization techniques, data augmentation and synthetic data generation have become key strategies for improving model robustness, especially in areas where collecting large-scale, high-quality datasets is costly or impractical [13]. Augmentation methods—such as random cropping, rotation, scaling, time jittering (for time series signals), and noise injection—help models generalize to different scenarios [14]. The dataset in this paper is not large in terms of data volume. In the case of large-scale high-quality datasets, more considerations must be taken into account during training.

Combining convolutional neural networks (CNNs), bidirectional long short-term memory networks (Bi-LSTMs), and attention mechanisms represents a powerful approach to modeling complex data with spatial and temporal features. When combined with sufficient data volume and carefully designed training strategies, these advanced architectures demonstrate clear performance advantages over simpler models. This progress highlights the ongoing evolution of deep learning research, with hybrid models continuously pushing the boundaries of predictive accuracy and application scope. Future research could explore integrating modules such as transformers, graph neural networks, or domain-specific feature engineering to further enhance model capabilities. Additionally, expanding dataset size and diversifying data sources holds promise for further improvements in generalization ability and robustness.

The confusion matrix in Figure 6 shows that the model performs well in recognizing dynamic behaviors, but there is symmetry confusion between static behaviors. This is consistent with the 2D feature distribution in the figure. 3D visualization further reveals the spatial structure—static behaviors exhibit layered separation along the *Z*-axis, indicating that the bidirectional LSTM successfully captures posture height features. These visualizations collectively demonstrate: (1) residual networks exhibit high discriminative power in extracting motion features; (2) static behavior classification performance is constrained by the continuity of action transitions; (3) attention mechanisms effectively focus on key time frames (e.g., the vertical posture maintenance phase of standing).

We also conducted comparative experiments with pure CNN [66] and pure LSTM [67], as well as our deep learning architecture. The comparisons, as given in Table 2, showed that compared to the baseline model, our deep learning architecture outperformed CNN and LSTM in accuracy. Furthermore, its recall and F1 score also performed well, making it a worthwhile architecture for implementing pose recognition tri-classification tasks.

### 3.3. Results on the Effect of Sensor Importance Under the Channel Attention Mechanism

**Figure 7:**
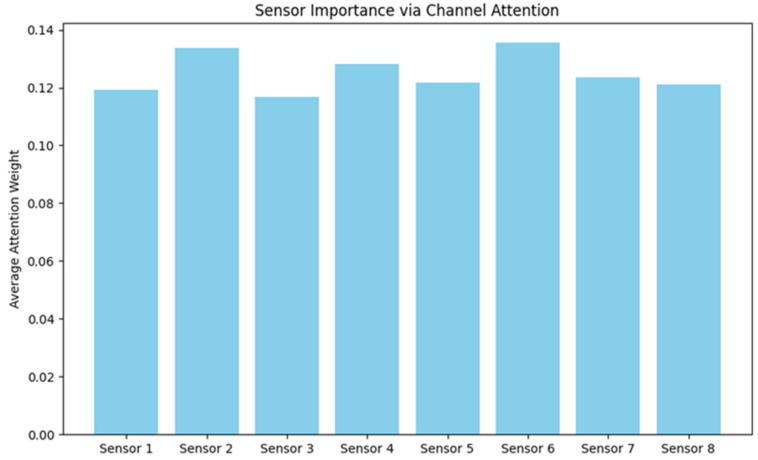
Average attention weight vs. distribution on individual sensors.

This channel attention mechanism handles structured numerical data by weighting the importance of multiple sensors’ features through the channel attention layer, and then the classification layer outputs the weighted features as classification probabilities for three categories to complete the sensor importance learning, and the model automatically determines which sensors are more important for the current classification task through the attention weights. From the results as given in Figure 7, 2, 4, and 6 sensors have better average attention weights for the overall array of 8 sensors, which is because the upper three rows of sensor elements are placed directly on the knee.

The above indicates that these sensors play a key role in behavioral recognition tasks. This adaptive sensor importance learning mechanism not only verifies the differences in the contribution of different sensor units to behavioral features but also provides data-driven theoretical basis for the optimization of sensor layout in wearable devices: in resource-constrained scenarios, high-weight sensors can be prioritized to maintain classification performance. Moreover, this mechanism enhances the interpretability of the recognition model by revealing the relationship between specific sensor placements and their corresponding biomechanical relevance during different motions. Such insights not only facilitate targeted hardware design, where redundant sensors can be reduced without significantly sacrificing accuracy, but also open avenues for personalized sensor deployment strategies tailored to individual gait characteristics or rehabilitation needs.

## 4. Conclusions

This paper presented a novel lightweight two-layer fabric strain sensor array tailored for comprehensive knee joint monitoring. There are three innovations: (1). The design of 8 sensors is more advantageous compared to single sensor or 2 × 2 sensors contact range. (2). Three experiments with different postures for triple classification of postures. (3). The use of two machine learning algorithms to improve the final results.

Based on these innovations, the proposed system achieved an encouraging balance between sensor hardware simplicity and analytical capabilities, providing a scalable framework for wearable motion monitoring. Firstly, the denser 8-sensor configuration enables more precise capture of strain distribution patterns in the knee region, thereby enhancing feature richness and improving classification accuracy, particularly in distinguishing subtle posture differences. Secondly, the inclusion of three distinct postures in the experimental protocol lays the groundwork for future expansion to more complex motion libraries, supporting richer activity recognition tasks. Further, the dual-algorithm approach not only provides comparative insights into model performance but also opens the door to hybrid or integrated models that can leverage the strengths of different classifiers to enhance robustness. Therefore, this fabric sensing system holds significant promise for future applications in rehabilitation monitoring, sports science analytics, and personalized healthcare within the medical and athletic domains.

Despite current limitations in pose diversity and generalization to unconstrained real-world movements, targeted applications for rehabilitation monitoring and movement performance optimization may still offer immediate deployment opportunities. Nonetheless, this limitation underscores the need for further research to expand the range of monitored activities and enhance the system’s robustness in diverse scenarios. For example, future research shall include more detailed experiments, such as investigating the effect of different speeds of the same movement [68], restricted movements [69], and differences in age [70]. In addition, future research could focus on integrating additional sensor modalities, expanding action datasets, and implementing real-time feedback systems to fully unlock the potential of knee-worn fabric strain sensors in personalized healthcare and sports training scenarios. By addressing current limitations and exploring new avenues, we anticipate that this technology will play a pivotal role in advancing personalized healthcare and sports training scenarios. 

## Figures and Tables

**Table 1:** Height, weight, and age information of the ten test subjects.

No.	1	2	3	4	5	6	7	8	9	10
Age	23	25	22	27	25	24	26	23	23	23
Height (cm)	183	182	173	174	167	192	178	177	174	174
Weight (kg)	76	95	80	75	74	86	77	70	67	70

**Table 2:** Comparison of experimental results for CNN, LSTM, and CNN + BiLSTM + Attention.

Models	CNN	LSTM	CNN + BiLSTM + Attention
Accuracy	0.79	0.67	0.95
Recall rate	0.77	0.66	0.94
F1 score	0.77	0.65	0.96

## Data Availability

The data presented in this study are available on request from the corresponding author. Data are not publicly available due to privacy and ethical reasons.
